# Neuronal and Brain Maturation

**DOI:** 10.3390/ijms23084400

**Published:** 2022-04-15

**Authors:** Luca Bonfanti, Sébastien Couillard-Després

**Affiliations:** 1 Neuroscience Institute Cavalieri Ottolenghi (NICO), 10043 Orbassano, Italy; 2 Department of Veterinary Sciences, University of Turin, 10095 Torino, Italy; 3 Spinal Cord Injury and Tissue Regeneration Center Salzburg (SCI-TReCS), Paracelsus Medical University, 5020 Salzburg, Austria; 4 Institute of Experimental Neuroregeneration, Paracelsus Medical University, 5020 Salzburg, Austria; 5 Austrian Cluster for Tissue Regeneration, 1200 Vienna, Austria

Can plasticity be considered as an extension of “immaturity”? Most forms of neural plasticity are retentions of “embryonic” aptitudes, some of which extend into postnatal ages [1]. Globally, such brain “immaturity” progressively decreases with age. Yet, each anatomical area has its own time windows of plasticity, which results in a mosaic of maturation degrees across brain regions and cell populations [2,3]. In addition, plastic changes vary remarkably among mammalian species according to their developmental schedules. All these aspects are integrating our previous view of neuronal maturation at the cellular level, thus becoming a theme that is promising new, exciting progress [4].

The aim of this special edition is to highlight the multifaceted aspects of brain structural plasticity, and to apply a special focus on aspects of “protracted immaturity”. The latter enables the nervous system to continue to grow postnatally (even throughout life in some regions) and to adapt the prototypic neural circuits to the surrounding environment based on individual experiences.

In recent years, the picture of brain plasticity and maturation has increased in complexity due to the discovery of new forms of plasticity and the identification of subtle “nuances” between its different forms [5]. This groundbreaking discovery more than thirty years ago of adult neurogenesis based on a population of neural stem cells in mammals has drawn much attention and eclipsed growing evidence on other ways neurons can maintain immaturity and plasticity despite the rather static (non-proliferative) adult brain [6]. We are discovering that the multifaceted aspects of plasticity can embrace a wide range of “types and scales,” with microscopic modifications affecting small portions of pre-existing cells (neurons, glia; e.g., synaptic plasticity) and more comprehensive changes varying at the level of cell numbers (adult neurogenesis, gliogenesis). Such emerging complexity is addressed in the review article by Bonfanti and Charvet [7] of this collection. Thus, we can reason in terms of neuronal (single cells) and brain (entire regions and/or cell populations) maturation with strong implications for brain development and developmental defects, prevention of a wide range of neurological states, and as potential therapeutic approaches.

Most articles in this Special Issue deal with the maturation of specific cell types and/or neuronal populations: the principal neurons of the primary motor cortex layer V [8], the non-newly generated, “immature” neurons in the piriform cortex [9], the GABA (γ-aminobutyric acid)-expressing inhibitory neurons of the cerebral cortex [10], the excitatory neurons of the hippocampal dentate gyrus [11], and the immature neurons of the paralaminar nucleus of the primate amygdala [12].

In addition, the functional modulation of neural precursors and their maturation has been addressed in different models: the neurogenic response of neural precursors of the meningeal niche after exposure of animals to an enriched environment [13]; the role for the brain-specific postsynaptic protein Neurogranin in the molecular mechanisms underlying granule olfactory cell plasticity and the formation of olfactory associative memory [14]; hypoxia as a driving force of demand-oriented neuroplasticity and the consequential “brain hardware upgrade” [15]; the significant alterations in the energy metabolism as possible cause for impaired function of the aged brain [16]; the effects of an early deviation from typical neurodevelopment resulting from the reduction in tuberous sclerosis complex (TSC) activity on the developing neural network, in the context of disease pathophysiology [17]; and finally how sex steroids can influence the organization of sexually dimorphic neural circuits underlying behaviors critical for survival and highly sensitive to external stimuli [18].

Two review articles are intended to make the point on the molecular mechanisms underlying plasticity and maturation, with special reference to the involvement of guidance cues from the Semaphorin and Plexin protein families in the refinement of neuronal circuits in the first place [19], followed by a tour d’horizon of the roles of Perineuronal nets, a condensed form of an extracellular matrix surrounding the soma and proximal dendrites of subsets of neurons [20] (Figure 1). These contributions analyze the role of Semaphorins, Plexins, and Perineuronal nets in the process of neuronal maturation and in neural development and refining of circuits, up to the synaptic plasticity and functional connectivity of important brain regions, such as the hippocampus and neocortex. In addition, deep analysis is provided of current knowledge on the role of Perineuronal nets in the regulation of the critical periods, as well as in the mature brain, in learning and memory, and during ageing. Both articles also address the role of such molecules in neurological diseases and the lessons we can learn from development.

Finally, a detailed analysis of cell proliferation carried out in whole mouse brains at different ages [21] elegantly uncovers the number of cell divisions taking place in the different brain compartments, revealing the specific rates and age-sensitivity of neurogenesis in the two adult neurogenic niches, and remarkably, its stability through ages and the amount of cell proliferation within the brain parenchyma.

## Figures and Tables

**Figure 1:**
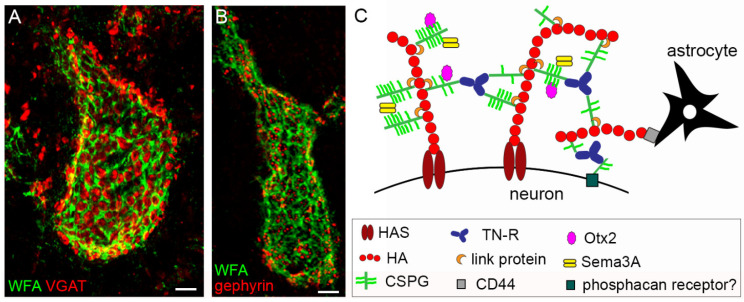
Structure and composition of the PNN. (**A**,**B**) show PNNs around neurons in the mouse cerebellar nuclei, labelled by Wisteria floribunda agglutinin (WFA), in green. PNNs display their typical holes, in which pre-synaptic terminals are contained. In (**A**), GABAergic terminals are shown (in red), labelled by anti-VGAT antibodies. In (**B**), post-synaptic clusters of gephyrin, which anchors GABA receptors to the underlying cytoskeleton, are shown (in red). In (**C**), the main molecular components of PNNs are depicted. Scale bar: 4 µm in (**A**,**B**). Image reproduced from Carulli and Verhaagen, with permission of MDPI, *Int. J. Mol. Sci*.

## References

[1] Urbàn N., Guillemot F. (2014). Neurogenesis in the embryonic and adult brain: Same regulators, different roles. Front. Cell. Neurosci..

[2] Lipp H.-P., Bonfanti L. (2016). Adult Neurogenesis in Mammals: Variations and Confusions. Brain Behav. Evol..

[3] Snyder J.S. (2019). Recalibrating the Relevance of Adult Neurogenesis. Trends Neurosci..

[4] Bradke F., Di Giovanni S., Fawcett J. (2020). Neuronal Maturation: Challenges and Opportunities in a Nascent Field. Trends Neurosci..

[5] König R., Benedetti B., Rotheneichner P., O’ Sullivan A., Kreutzer C., Belles M., Nacher J., Weiger T.M., Aigner L., Couillard-Després S. (2016). Distribution and Fate of DCX/PSA-NCAM Expressing Cells in the Adult Mammalian Cortex: A Local Reservoir for Adult Cortical Neuroplasticity?. Front. Biol..

[6] Bonfanti L., Seki T. (2021). The PSA-NCAM-Positive “Immature” Neurons: An Old Discovery Providing New Vistas on Brain Structural Plasticity. Cells.

[7] Bonfanti L., Charvet C.J. (2021). Brain Plasticity in Humans and Model Systems: Advances, Challenges, and Future Directions. Int. J. Mol. Sci..

[8] Benedetti B., Dannehl D., Janssen J.M., Corcelli C., Couillard-Després S., Engelhardt M. (2020). Structural and Functional Maturation of Rat Primary Motor Cortex Layer V Neurons. Int. J. Mol. Sci..

[9] Coviello S., Benedetti B., Jakubecova D., Belles M., Klimczak P., Gramuntell Y., Couillard-Despres S., Nacher J. (2021). PSA Depletion Induces the Differentiation of Immature Neurons in the Piriform Cortex of Adult Mice. Int. J. Mol. Sci..

[10] Kim J.-Y., Paredes M. (2021). Implications of Extended Inhibitory Neuron Development. Int. J. Mol. Sci..

[11] Annamneedi A., del Angel M., Gundelfinger E., Stork O., Çalışkan G. (2021). The Presynaptic Scaffold Protein Bassoon in Forebrain Excitatory Neurons Mediates Hippocampal Circuit Maturation: Potential Involvement of TrkB Signalling. Int. J. Mol. Sci..

[12] Chareyron L.J., Banta Lavenex P., Amaral D.G., Lavenex P. (2021). Life and Death of Immature Neurons in the Juvenile and Adult Primate Amygdala. Int. J. Mol. Sci..

[13] Zorzin S., Corsi A., Ciarpella F., Bottani E., Dolci S., Malpeli G., Pino A., Amenta A., Fumagalli G.F., Chiamulera C. (2021). Environmental Enrichment Induces Meningeal Niche Remodeling through TrkB-Mediated Signaling. Int. J. Mol. Sci..

[14] Gribaudo S., Saraulli D., Nato G., Bonzano S., Gambarotta G., Luzzati F., Costanzi M., Peretto P., Bovetti S., De Marchis S. (2021). Neurogranin Regulates Adult-Born Olfactory Granule Cell Spine Density and Odor-Reward Associative Memory in Mice. Int. J. Mol. Sci..

[15] Butt U., Hassouna I., Garcia-Agudo L.F., Steixner-Kumar A., Depp C., Barnkothe N., Zillmann M., Ronnenberg A., Bonet V., Goebbels S. (2021). CaMKIIα Expressing Neurons to Report Activity-Related Endogenous Hypoxia upon Motor-Cognitive Challenge. Int. J. Mol. Sci..

[16] Gostomska-Pampuch K., Drulis-Fajdasz D., Gizak A., Wiśniewski J., Rakus D. (2021). Absolute Proteome Analysis of Hippocampus, Cortex and Cerebellum in Aged and Young Mice Reveals Changes in Energy Metabolism. Int. J. Mol. Sci..

[17] Bassetti D., Luhmann H., Kirischuk S. (2021). Effects of Mutations in TSC Genes on Neurodevelopment and Synaptic Transmission. Int. J. Mol. Sci..

[18] Trova S., Bovetti S., Bonzano S., De Marchis S., Peretto P. (2021). Sex Steroids and the Shaping of the Peripubertal Brain: The Sexual-Dimorphic Set-Up of Adult Neurogenesis. Int. J. Mol. Sci..

[19] Limoni G. (2021). Modelling and Refining Neuronal Circuits with Guidance Cues: Involvement of Semaphorins. Int. J. Mol. Sci..

[20] Carulli D., Verhaagen J. (2021). An Extracellular Perspective on CNS Maturation: Perineuronal Nets and the Control of Plasticity. Int. J. Mol. Sci..

[21] Semenov M. (2021). Proliferative Capacity of Adult Mouse Brain. Int. J. Mol. Sci..

